# Supplementary material for: Prenatal Workshops and Support Groups for Prospective Parents Whose Children Will Need Neonatal Care at Birth: A Feasibility and Pilot Study
Source: Children (Basel). 2023 Sep 19;10(9):1570. doi: 10.3390/children10091570 (PMC10529479; doi:10.3390/children10091570)

SUPPLEMENTARY MATERIAL

S1. DOCUMENTS THAT CAN BE SENT TO PARENTS VIA EMAIL:
(Ask who would like to receive the documents and write down the emails) Suggested email:
Dear [name of parents that were met],
It was a pleasure to talk about your baby boy/girl with you.

As promised, here are the documents that are appreciated by parents when their baby is in the NICU:

-A virtual visit of the unit
https://vimeo.com/449839052/d8864348
0b

-The admission guide for the NICU
https://www.chusj.org/getmedia/8d373e06-b655-4c0d-85c8-d2fd38fadcea/F-2814_guide-de- vie_Neonatologie.pdf.aspx?ext=.pdf

-The procedure to express breastmilk after birth
-La procédure pour exprimer le lait maternel après la naissance avec le tire-lait professionnel (qui se trouvent à la salle d'accouchement et en néonatologie)
https://vimeo.com/430113851/a3abcaa307

-A short video for NICU parents (Survival guide for parents in the NICU) https://www.youtube.com/watch?v=U6fA9QNqCT4

You can also come back to the workshop every Friday, Good luck for the rest of your pregnancy,
[Your name]

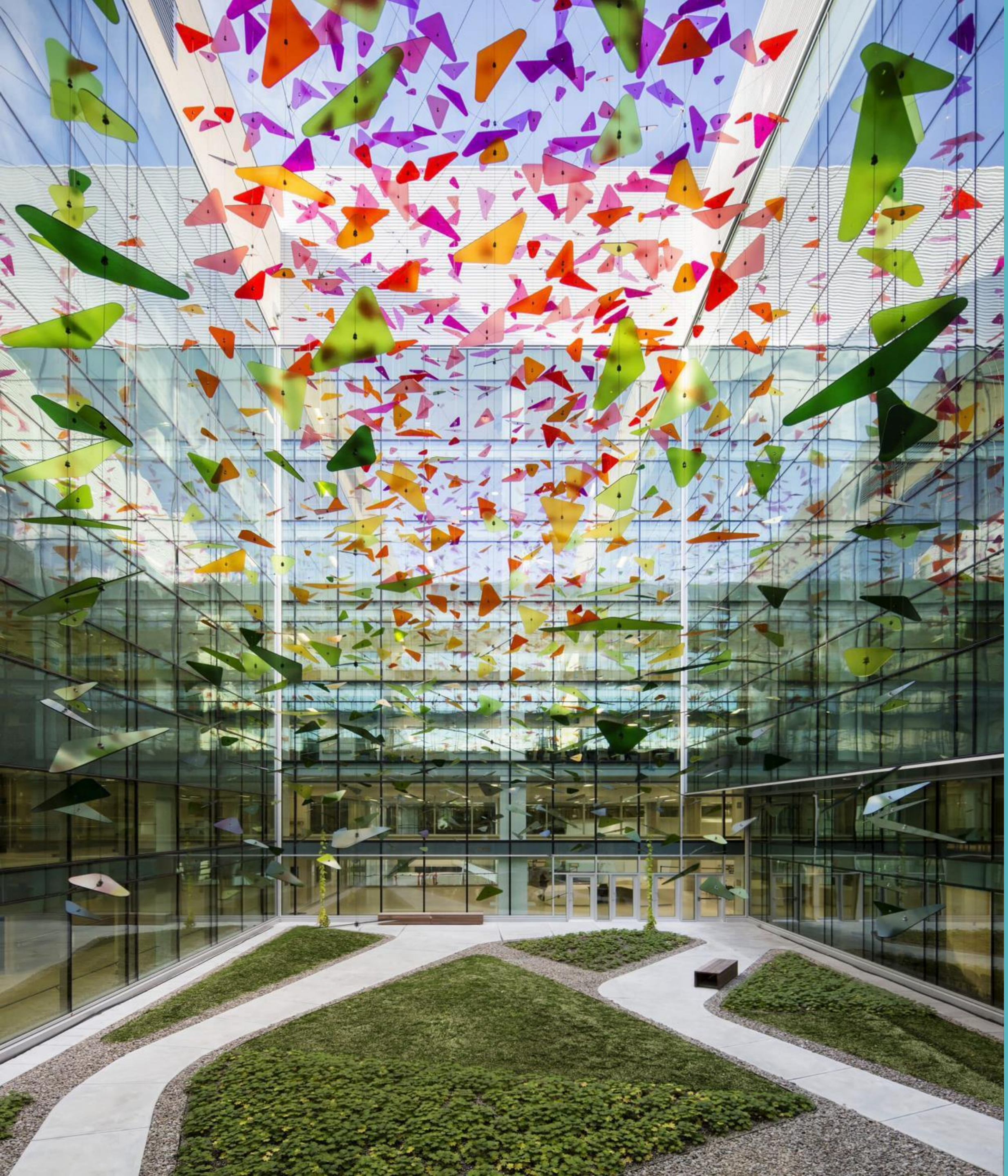

PRENATAL NEONATAL WORKSHOP

CHU Sainte-Justine
Le centre hospitalier universitaire mère-enfant

Université de Montréal

Presentations

- It is hard to be hospitalized on the high-risk pregnancy unit or to know that your baby will be hospitalized in the NICU
- Parents in your situation go through many emotions
  - Sadness, stress, anxiety
  - Anger
  - Guilt (It is NOT your fault! There is NOTHING you

could have done to prevent what is happening to you and your baby)

- Isolation

Neonatology

For babies who need help at birth

- Babies that need surveillance or care
- At CHU Sainte-Justine: 5th floor bloc 11
  - Single or twin rooms
  - Virtual visit on video available
- Nursery (Stable babies and late preterm babies).

- Other hospitals

Parents in the NICU

- **All parents and all babies are different.**
- Babies are admitted either to intensive or intermediate care (according to the need of respiratory support).
- **You are welcome 24/7.**
- **Your presence is encouraged as often and as long as you want, according to your own**

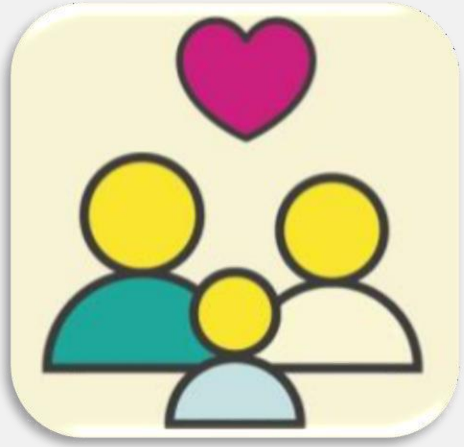

situation.

THE MACHINES

Parents that had a baby in neonatology shared they would have liked to know the machines around their baby before birth.

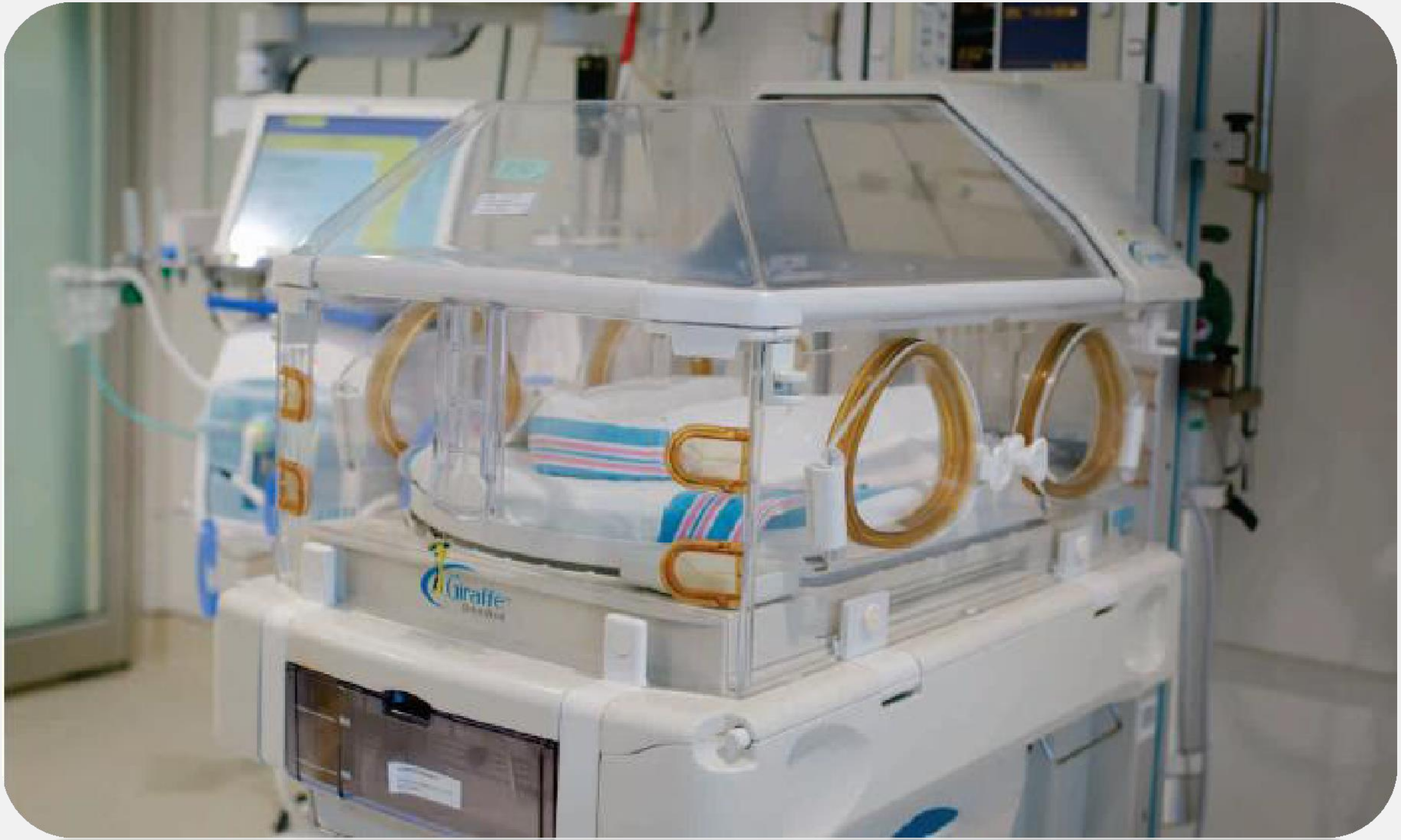

Cardiorespiratory monitor and the ALARMS

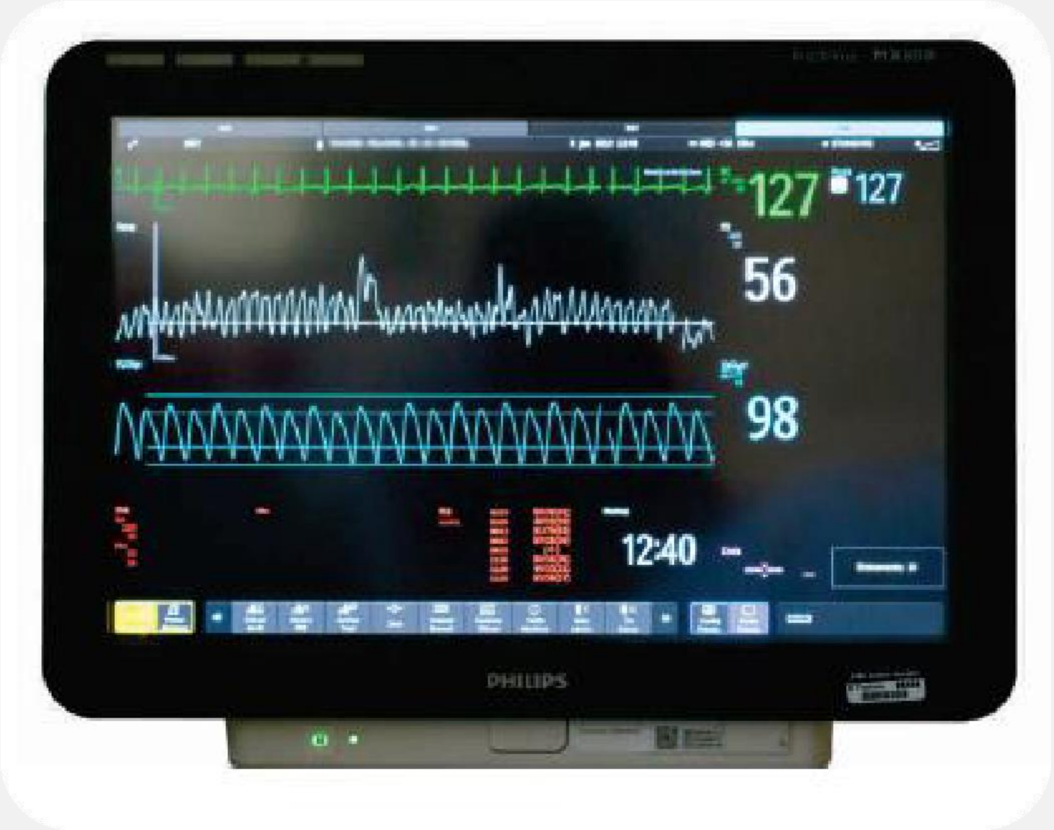

Feeding tube (naso-gastric tube)

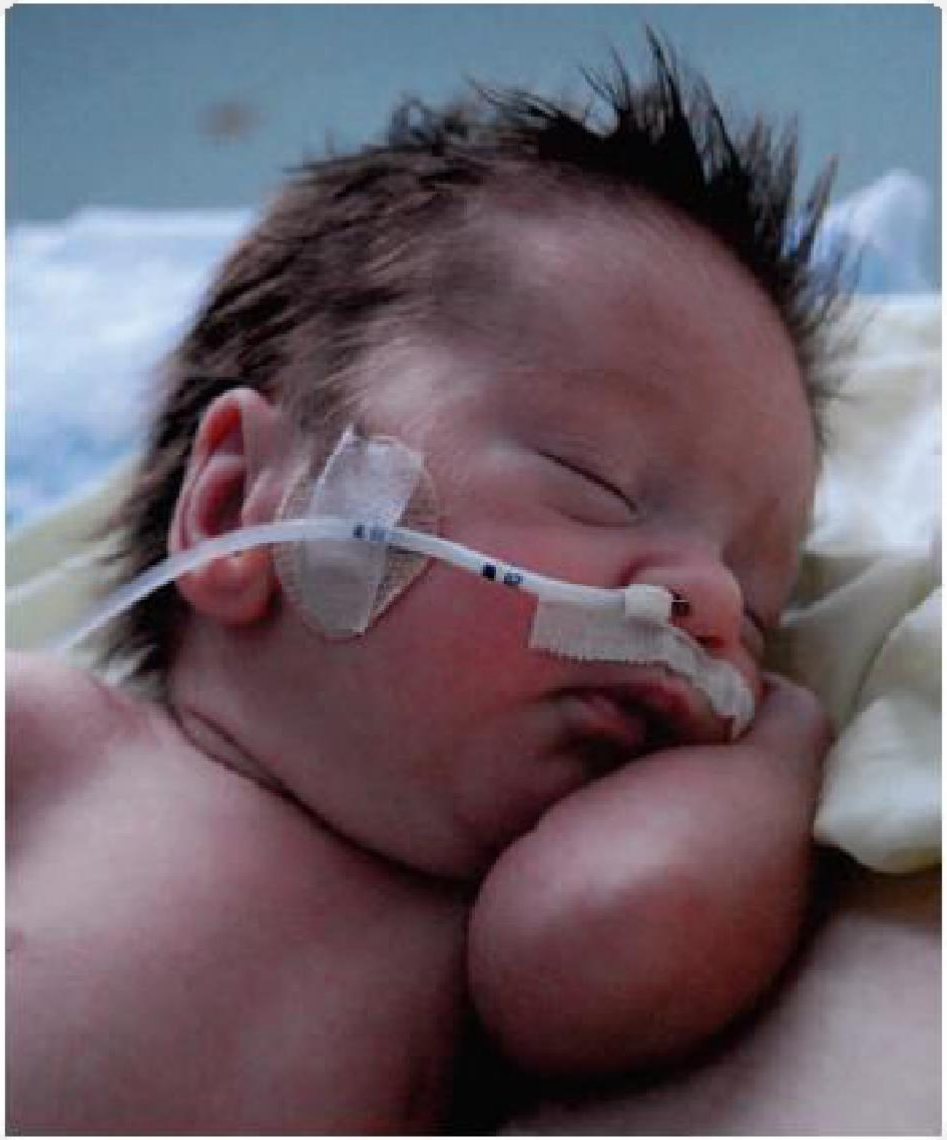

FEEDING

- Breastfeeding is possible
- Expressing breastmilk
  - Lactation consultant
  - Pump available in every room and a kit is provided
  - Expression guide: video also available
- Donor milk is also available (awaiting mother's milk or

for those who do not wish or cannot breastfeed)

Nasal cannulae

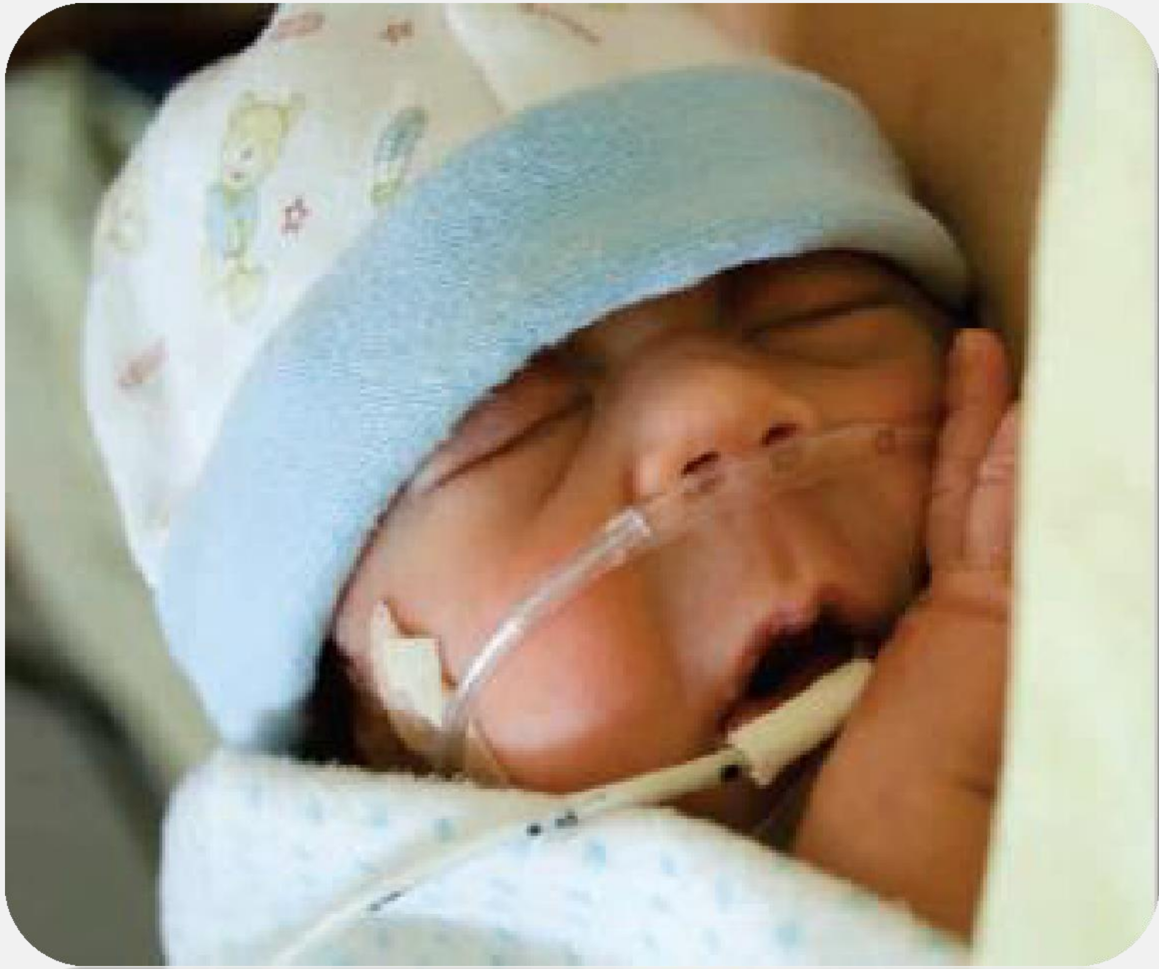

CPAP

(Continous Positive

Airway Pressure)

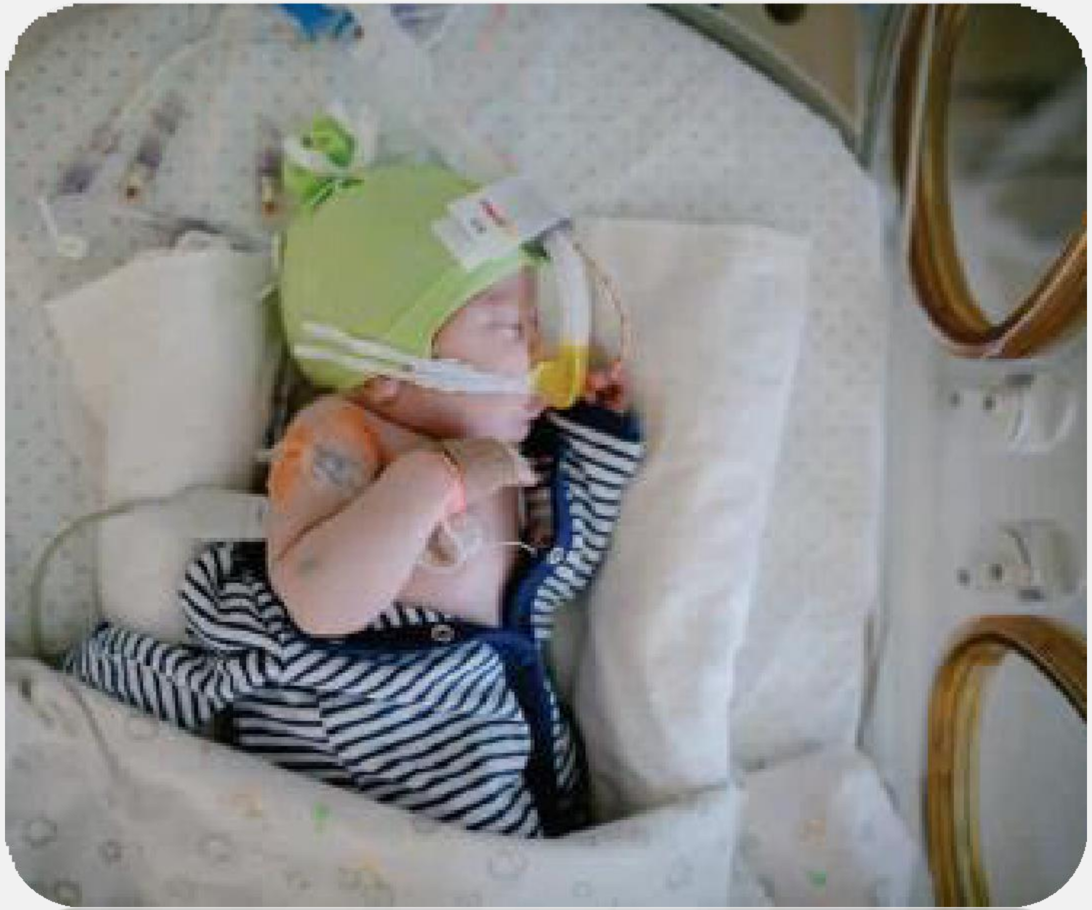

Intubation and endotracheal tube (ET Tube)

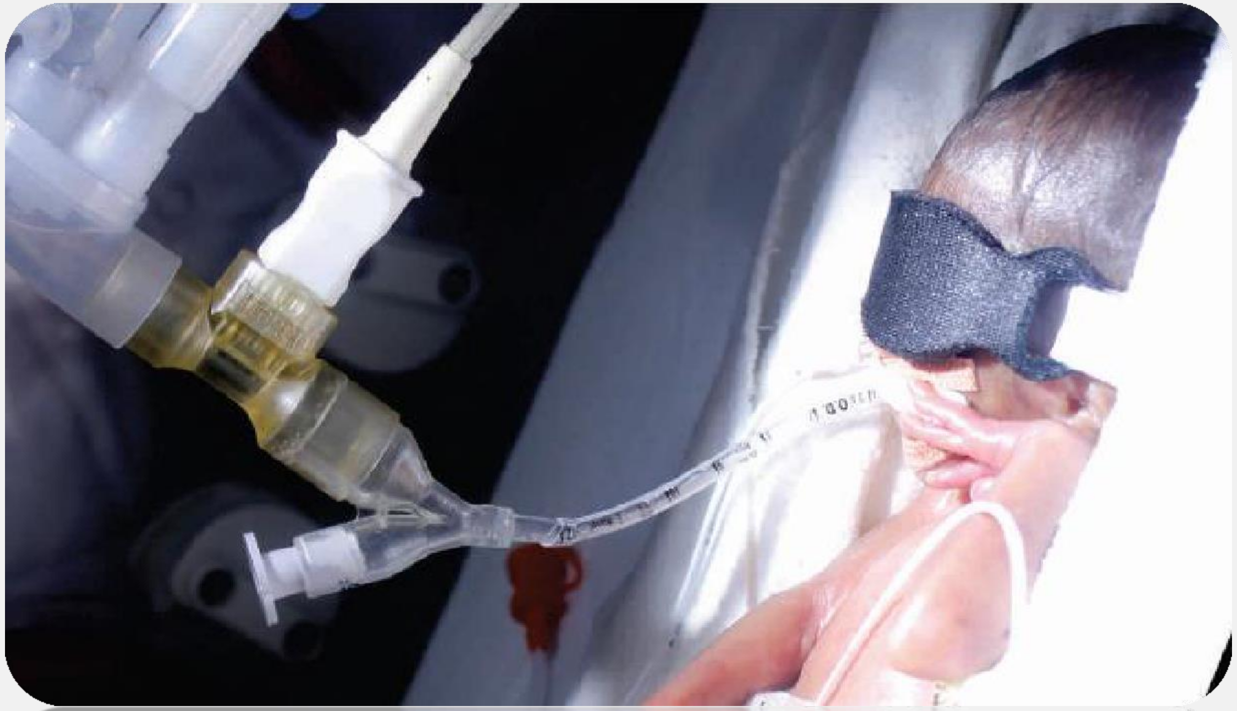

Parents’ role

- **You are essential!**
- The NICU is complicated
- Accumulation of griefs and losses
- It is a great challenge to learn to be a parent in neonatology and at the same time learn how to help your baby
- Go at your own pace
- Support team is available: psychologists, social workers,

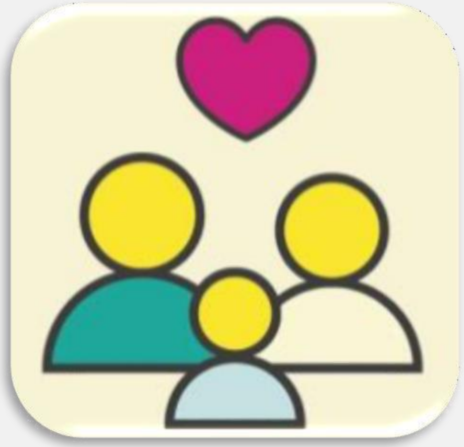

spiritual advisor

- **You are part of the team.**

How can parents participate?

- The first days are usually difficult.
- If mom is not able to come see her baby during the first days, pictures can be taken. You can place pictures of the family close to your baby.
- Parents, families and babies learn about each other every day.

Parents are essential

- **Even if your baby is small or fragile, you can touch and care for him/her:** change diaper, bathe, apply cream, change leads, etc.
- It is important your baby hears your voice, it helps develop the brain and the senses. Speak gently, use the baby's name, sing songs, read him a book (reading and music program).
- Other senses that are important: smell and touch
  - Holding your baby (skin-to-skin), speaking to your

baby, rocking, etc: all help brain development.

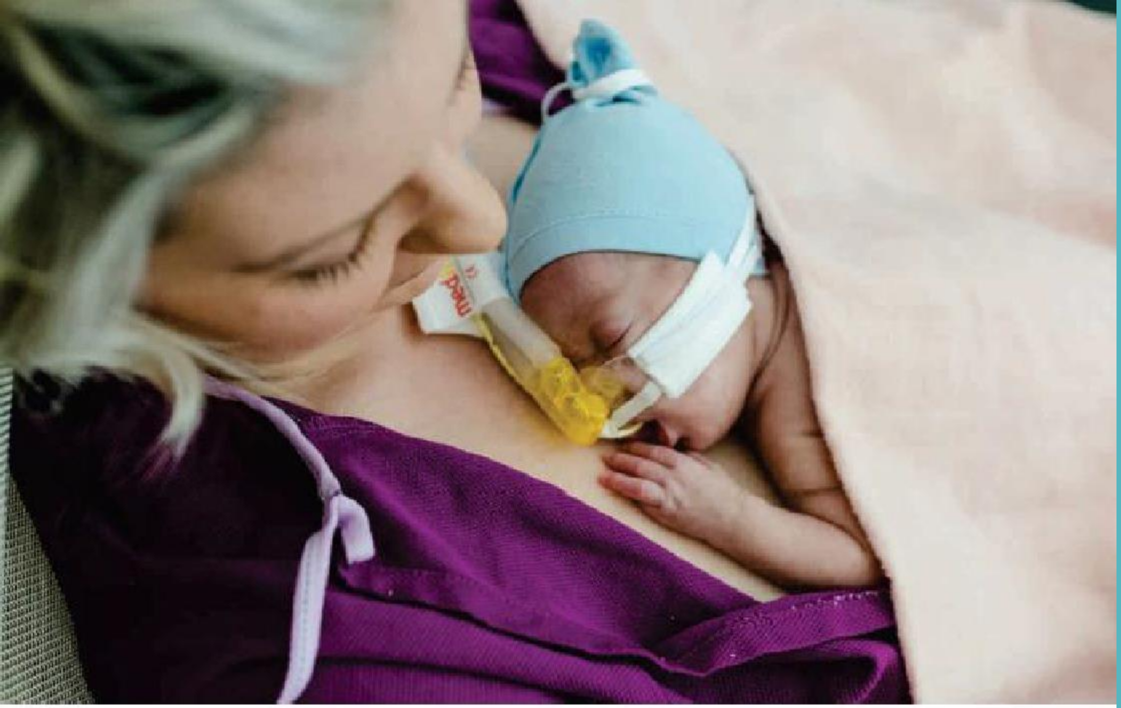

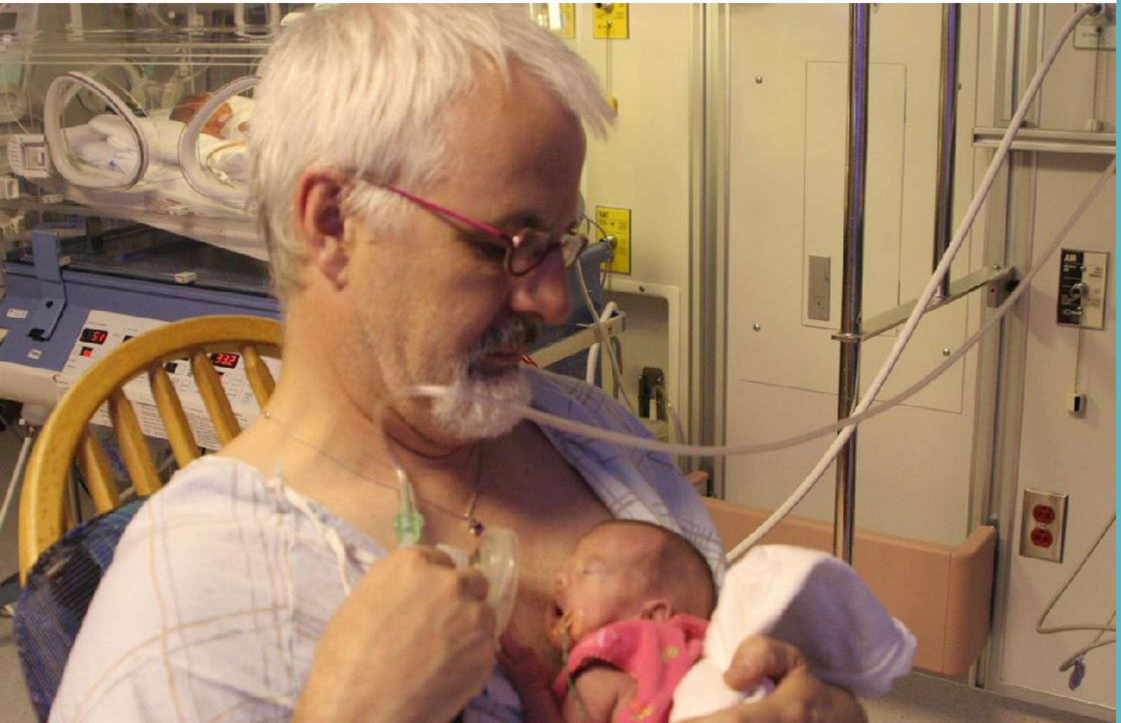

KANGAROO CARE (SKIN-TO-SKIN)

-For baby: pleasant sensations that resemble those experienced in the womb. And improves stability

-For moms: helps milk production.

-For parents: sometimes, for some parents there is some anxiety at the beginning, but this special moment is usually

Medical rounds

- Every day, during which medical decisions are taken.
- Parents are always welcome
- Between 10 h et 15 h, you can ask your nurse if you would like to be present.

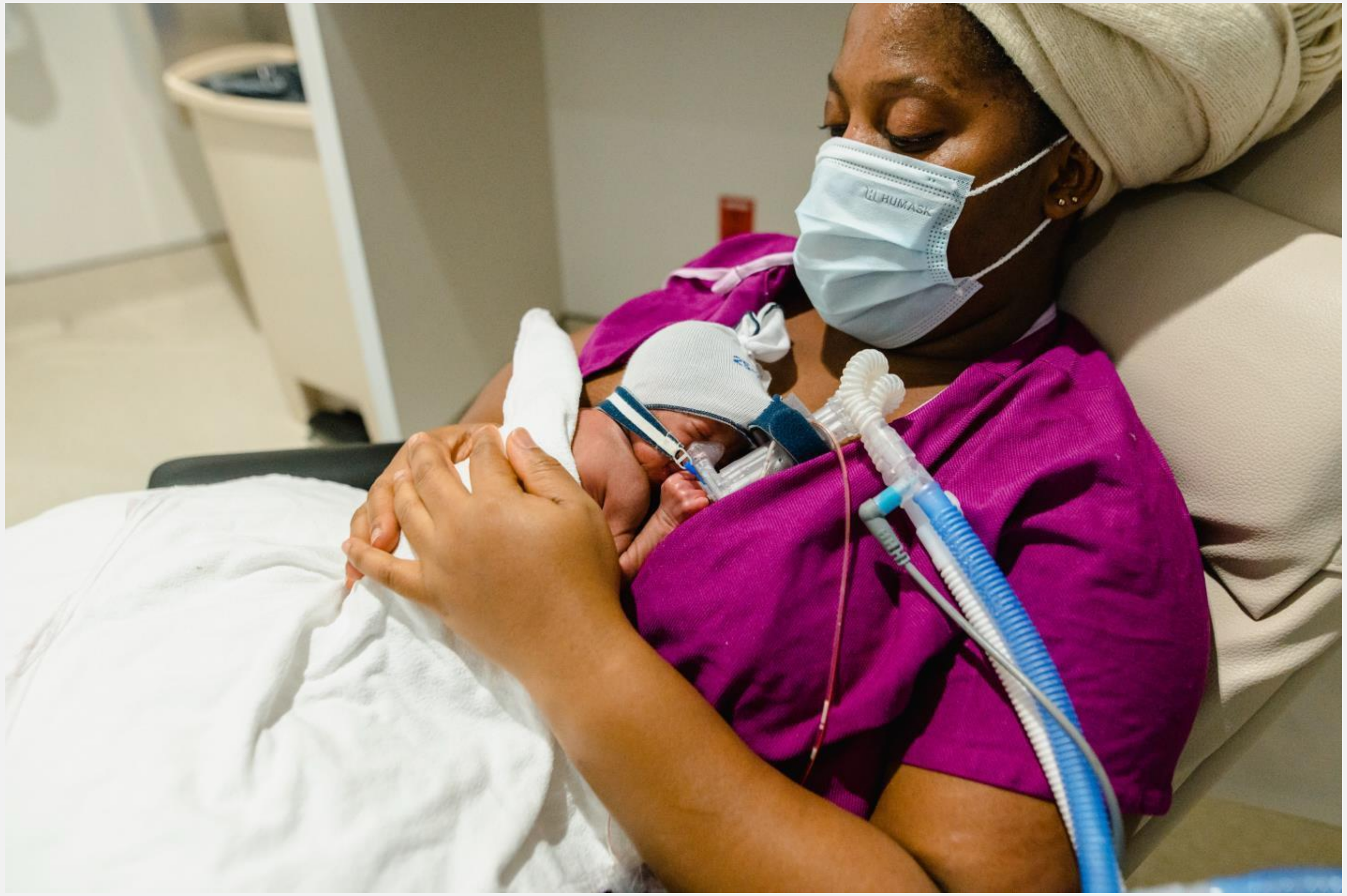

A white board is available in every room: it is a communication tool between you and clinicials

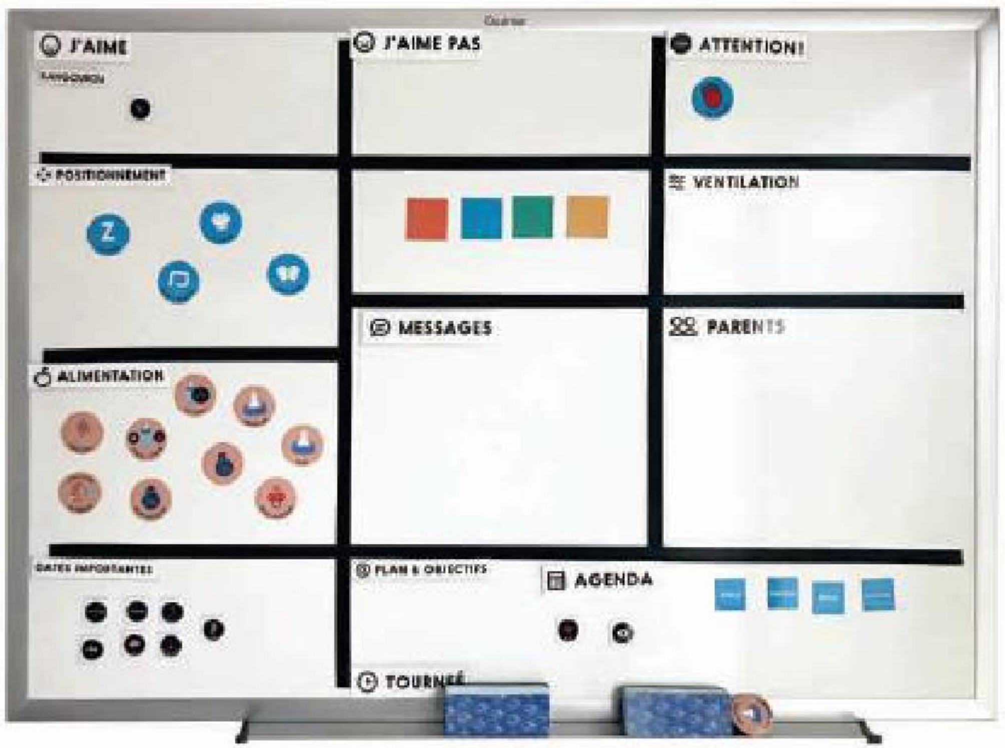

Advice

Write down every question to make sure

you don’t forget any

Nights

- **Pull-out couch** available in every room.
- Many parents choose to sleep at home. **It is important to get some rest/strength.**
- Temporary accommodation services available for families:

1. **In the hospital. Hôtellerie Chez Cachou**
2. **Close to the hospital. Manoir Ronald McDonald**

DISCHARGE FROM THE NICU

- Some babies can be transferred to a center closer to home
- Discharge home when:
  - Breathes alone with no pauses
  - Feeds on his own
  - Maintains a good body temperature
  - Not always on the due date, often before

Questions?

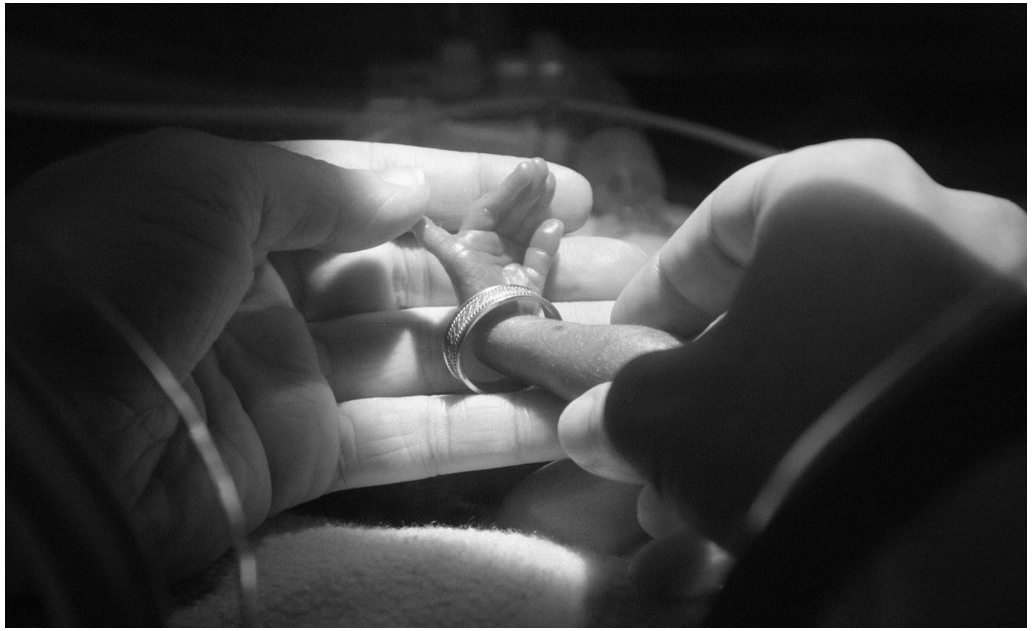

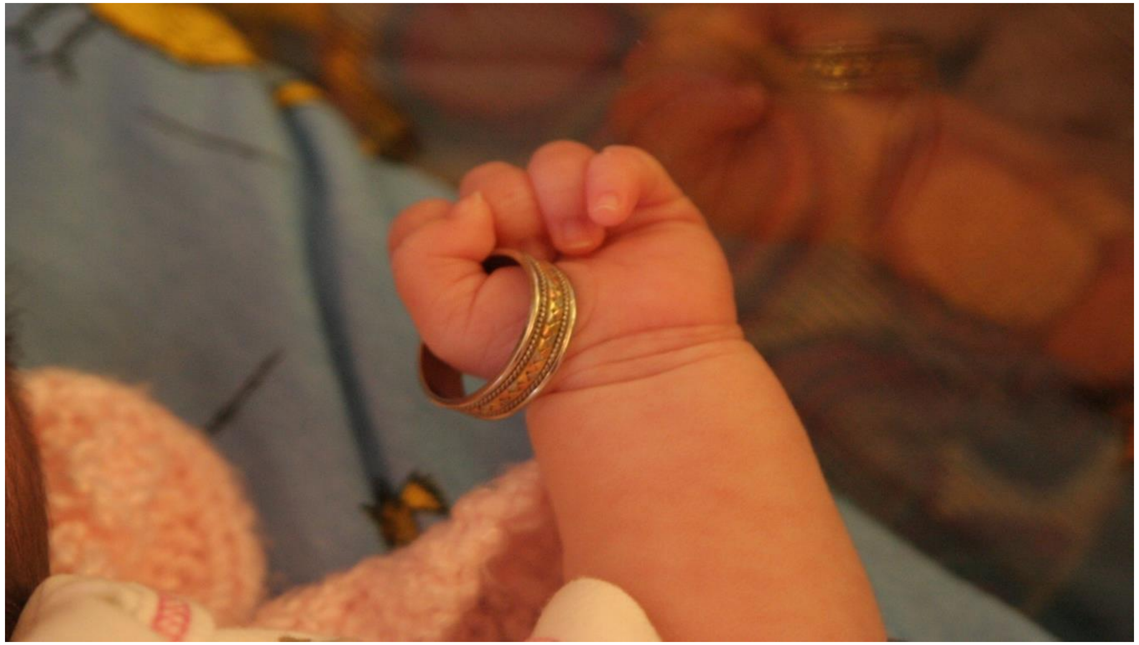

S2. OMBRELLE NÉONATOLOGIE: checklist de l'animateur

Avant l'atelier, rencontrer l'assistante infirmière chef de l'étage de grossesse à risque élevé (GARE):

- ☐ L'atelier a bien lieu? Combien de personnes ?
- ☐ Qui sera l'infirmière présente en « backup »?
- ☐ Aide (ou pas) pour installer certaines patientes de GARE dans la

salle ? <u>(Le powerpoint suit ces étapes, le checklist est en soutien)</u>

□ **Présentation : je suis qui, mon rôle en néonatologie, « médecine pour les bébés malades».**
□ **Confidentialité: on ne partage pas les histoires et ce qui se dit dans cette rencontre**
□ **Ce n'est pas parce que vous êtes à cet atelier que votre bébé va automatiquement aller en néonatologie**
□ **« tour de table » : qui est dans la salle, notre nom, celui de notre bébé et condition du bébé. OK si on ne veut pas dire notre nom ou se présenter.**

Normaliser les émotions des parents
□ On n'entend généralement pas parler de néonatologie dans la vie de tous les jours.
□ Normal d'être inquiets, incertitude.
□ L'incompréhension est normale, l'injustice par rapport à toutes celles qui ont des grossesses normales.
□ Culpabilité est fréquente mais pas constructive : Quand on est un papa et une maman qui aime notre enfant, notre bébé, quand quelque chose arrive, on se demande ce qu'on aurait pu ou du faire mieux. Ce n'est pas de votre faute, rien que vous auriez pu faire pour prévenir cela.
□ Avoir de la compassion envers vous-même : pas facile de rester isolées comme cela, pour le bien de votre bébé. Demande beaucoup de force. Vous êtes plus forte que vous le pensez
□ Impuissance mais, apprendre sur la néonatologie permet à bien des parents de se préparer

BUT: Parler de la néonatologie, répondre à vos questions, vous n'êtes pas seuls dans cette situation Voici ce que les parents veulent généralement savoir

QUESTIONS ?
□ Chambre du bébé : unique ou double
□ Stressant de voir son bébé branché de partout
□ Bien des alarmes, sonnent pas seulement quand il y a une urgence, les infirmières sont là

□ **Les parents sont les bienvenus 24h/24 7 jours/7**
□ Les parents font partie de l'équipe
□ Un bébé, même très prématuré : sent, entend bien, reconnait votre voix, ressent le toucher. Ils ne voient pas très bien. Les parents peuvent stimuler ces sens pour aider leur bébé, les infirmières sont là pour vous guider.
□ Peau-à-peau/Kangourou.
□ Routine avec l'infirmière : changer bébé, lui parler, le bain, etc.

□ CONGÉ : pas nécessairement à la date prévue d'accouchement ; bébés doivent manger et respirer seuls et garder leur température. Il arrive que les bébés partent 3 semaines avant leur date prévu d'accouchement, mais aussi plus tard. Rebours vers le domicile possible.

□ **Autre trucs que les parents de néonat /GARE auraient aimé savoir quand ils étaient dans vos souliers**
□ Choisir un porte-parole : épuisant de répondre toujours aux mêmes questions
□ Choisir l'entourage que l'on veut: normal de vouloir être seuls et de ne pas vouloir répondre au téléphone
□ Dire à nos proches exactement ce dont on a besoin : par exemple, apporter de la nourriture pour les enfants à la maison
□ Développer une routine en pensant à se reposer et faire des activités qui nous aident
□ Se concentrer sur ce qu'on contrôle, reconnaitre ce qu'on ne contrôle pas
□ Se féliciter tous les jours

DOCUMENTS QUI PEUVENT ÊTRE ENVOYÉS AUX PARENT PAR COURRIEL :
(demander qui voudrait recevoir ces documents et prendre les courriels)

Courriel suggéré :
Bonjour [nom des parents que l'on a rencontré],
Cela m'a fait plaisir de parler de votre fille avec vous.

Nous avons plusieurs documents qui sont appréciés par les parents de néonatologie et ceux qui se trouvent dans votre situation. Tel que promis voici :

-La vidéo de présentation de notre unité et une visite virtuelle de notre unité https://vimeo.com/449839052/d88643480b

-Le guide d'admission pour les parents de néonatologie (« Bienvenue en néonatologie ») https://www.chusj.org/getmedia/8d373e06-b655-4c0d-85c8-d2fd38fadcea/F-2814_guide-de- vie_Neonatologie.pdf.aspx?ext=.pdf
À noter que dans ce guide, les règles d'unité à propos des visiteurs qui peuvent venir voir les bébés changent régulièrement selon la situation COVID, donc cette page ne représente généralement pas ce qui se passe sur l'unité depuis le début de la pandémie. Les parents par contre sont et ont toujours été les bienvenus sur l'unité.

-La procédure pour exprimer le lait maternel après la naissance avec le tire-lait professionnel (qui se trouvent à la salle d'accouchement et en néonatologie)
https://vimeo.com/430113851/a3abcaa307

-Une petite vidéo faite pour les parents de néonatologie (Top 10 du guide de survie)
https://www.youtube.com/watch?v=U6fA9QNqCT4

Vous pouvez aussi revenir à l'atelier Ombrelle tous les vendredis, Bonne chance pour la fin de la grossesse,
[votre nom]

2

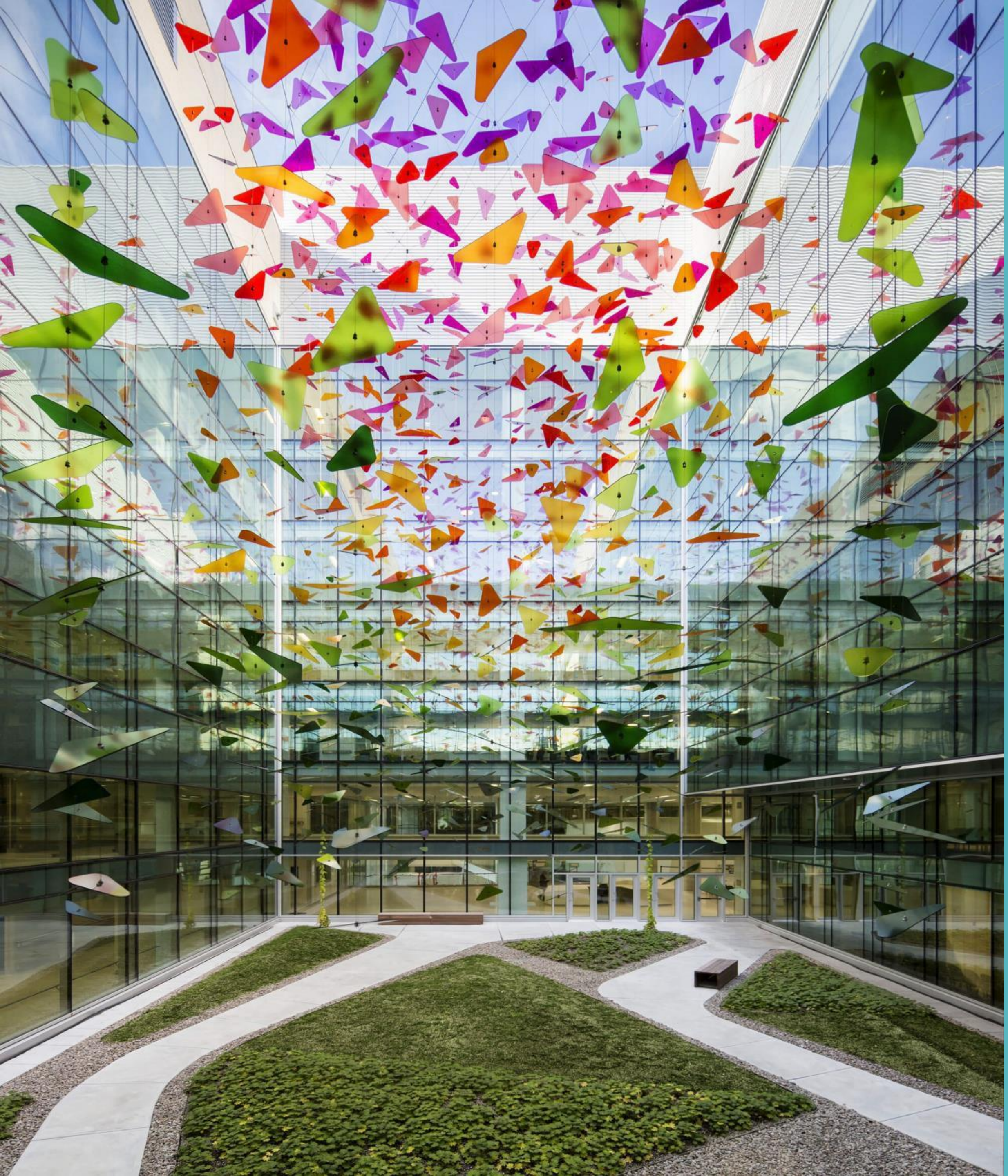

BIENVENUE EN NÉONATALOGIE
ATELIER PRÉNATAL

CHU Sainte-Justine
Le centre hospitalier universitaire mère-enfant
Université de Montréal

Présentations

- Pas facile d'être en Grossesse à risque Élevé (GARE) ou d'être parent d'un bébé qui ira en néonatologie
- Plusieurs émotions sont vécues par les parents dans votre situation
  - Tristesse, stress, anxiété
  - Colère
  - Culpabilité (non, ce n'est pas de votre faute! Vous n'auriez rien pu faire pour éviter ce qui arrive à votre bébé)
  - Isolement

La néonatologie

Bébés qui ont besoin d'aide à la naissance

- Bébés qui ont besoin de surveillance ou de soins
- Au CHU Sainte-Justine: 5ème bloc 11, chambres uniques et doubles
  - Chambres individuelles ou de jumeaux
  - Vidéo disponible où les chambres sont montrées
- La pouponnière (bébés plus stables et moins prématurés)
- Les autres centres

La place des parents en néonatologie

- **Tous les parents sont différents, tous les bébés sont différents.**
- Les bébés sont admis aux soins intermédiaires ou aux soins intensifs (selon leur besoin de soutien respiratoire).
- **Vous êtes bienvenus en néonatologie 24h sur 24.**
- **Votre présence est encouragée aussi souvent et longtemps que vous le désirez, selon votre réalité.**

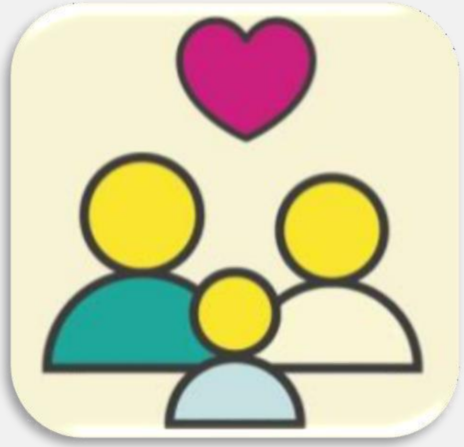

LES MACHINES

Les parents qui sont passés par la néonatologie nous ont partagé qu'ils auraient aimé connaître les machines autour de

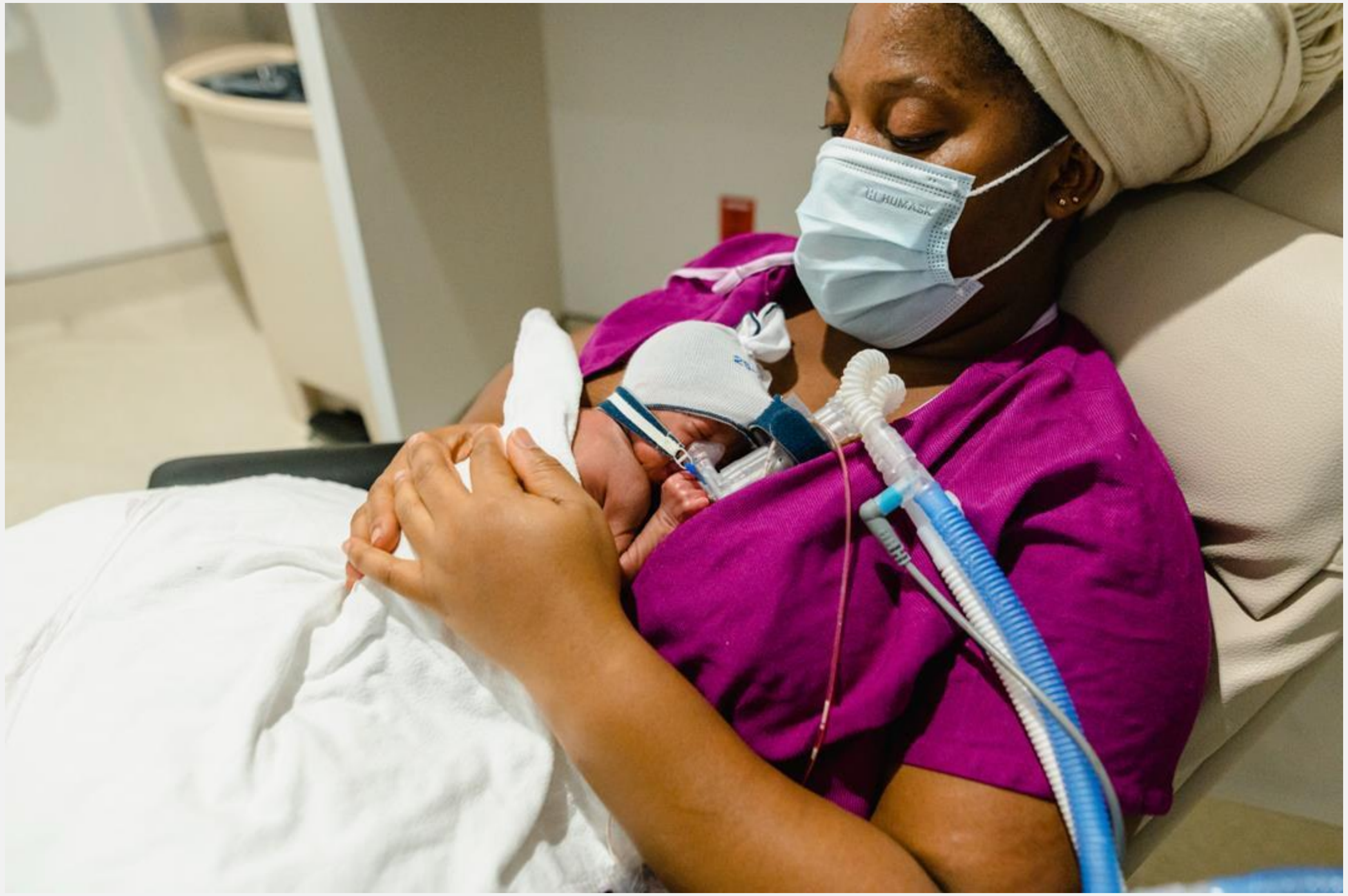

leur bébé avant sa
naissance

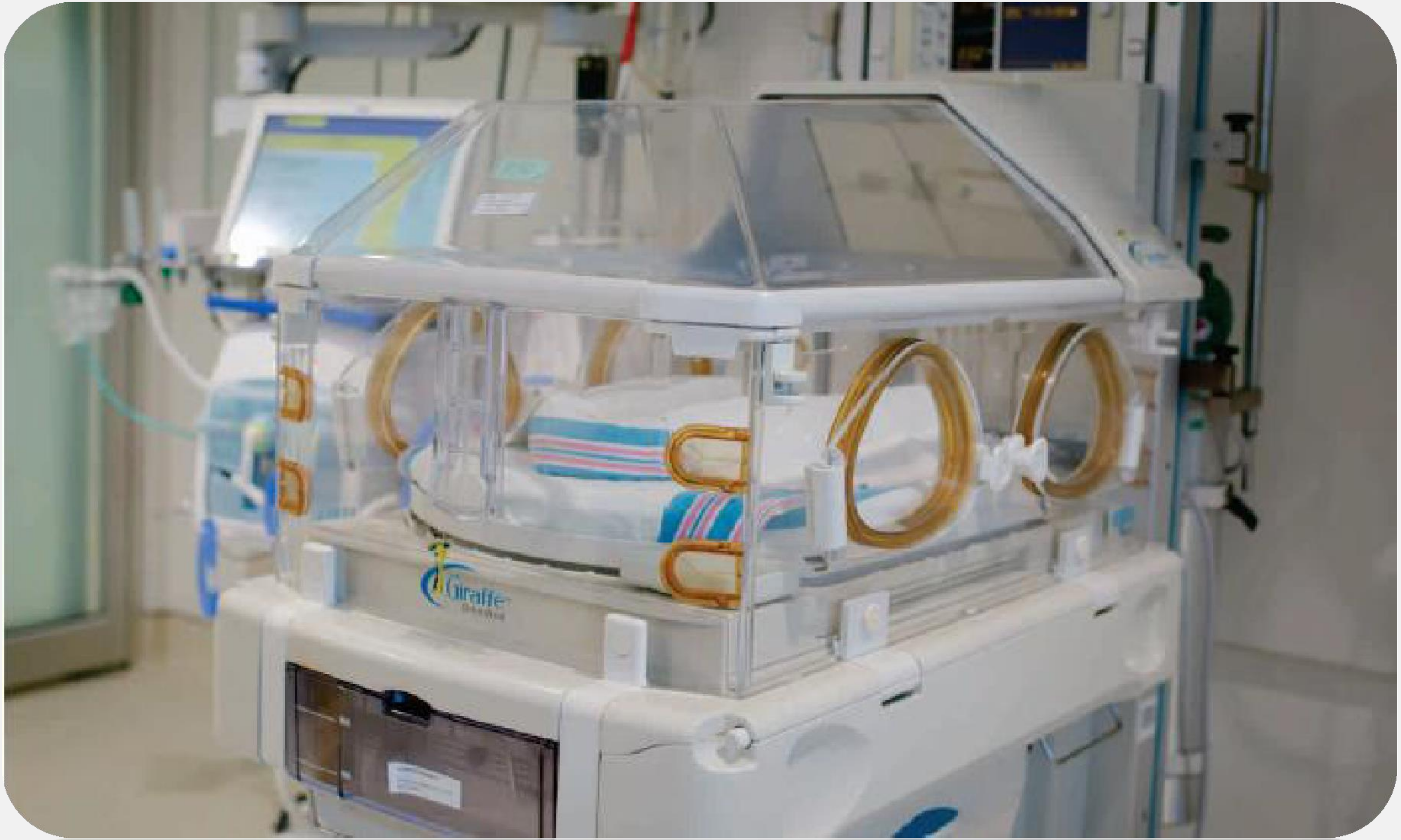
Giraffe

Moniteur cardiorespiratoire et LES ALARMES

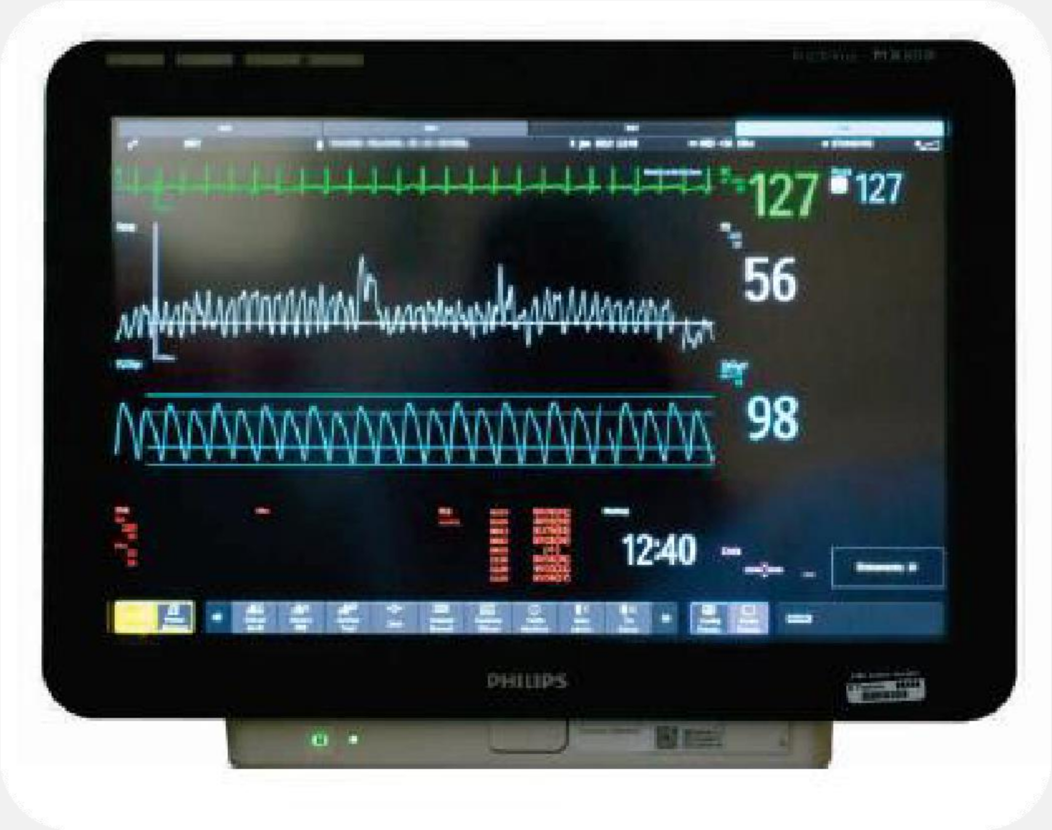

Tube de « gavage »
(naso-gastrique)

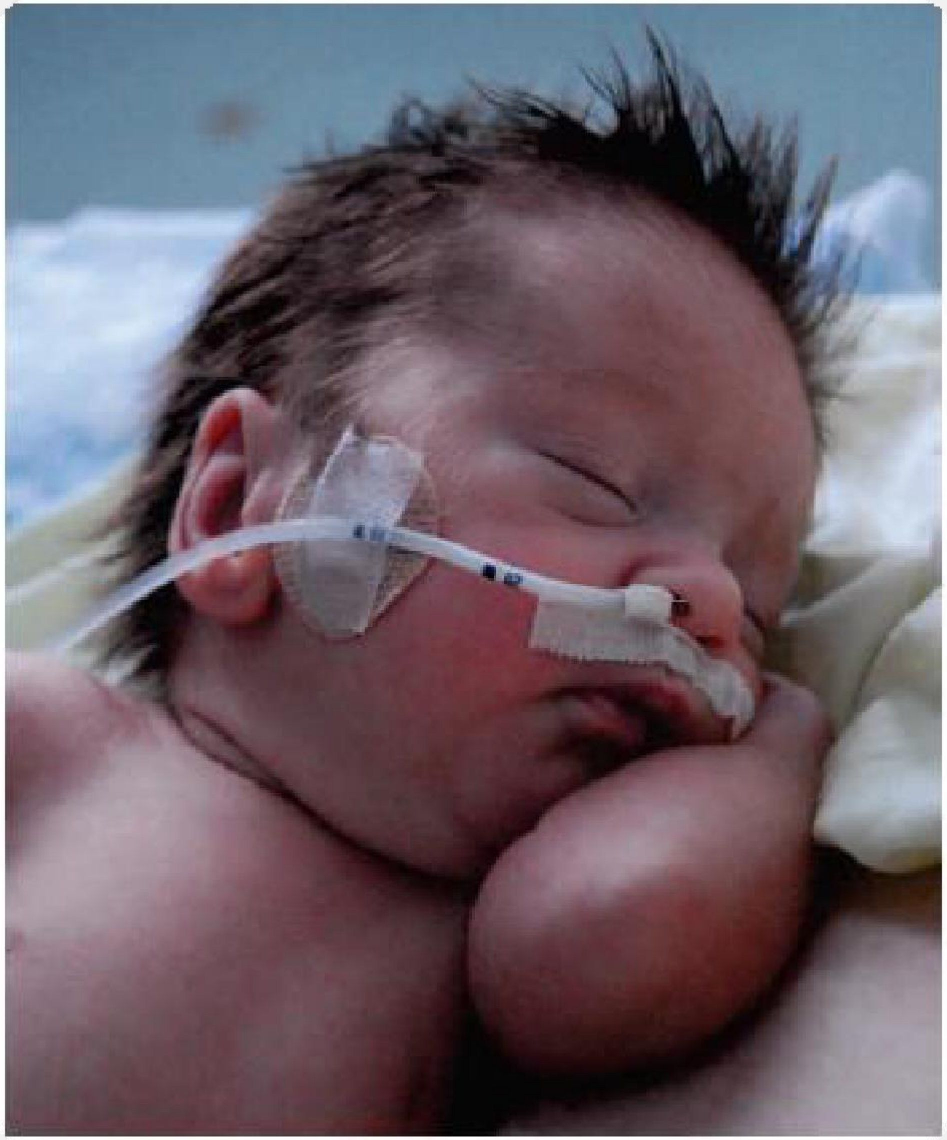

L'ALIMENTATION

- Allaitement possible
- Expression du lait
  - Conseillères en lactation
  - Tire lait dans toutes les chambres et kit de tire-lait fourni
  - Guide d'expression: vidéo disponible
- Lait de banque en attendant le lait de maman ou pour celles

qui ne veulent ou ne peuvent pas allaiter

Lunettes nasales

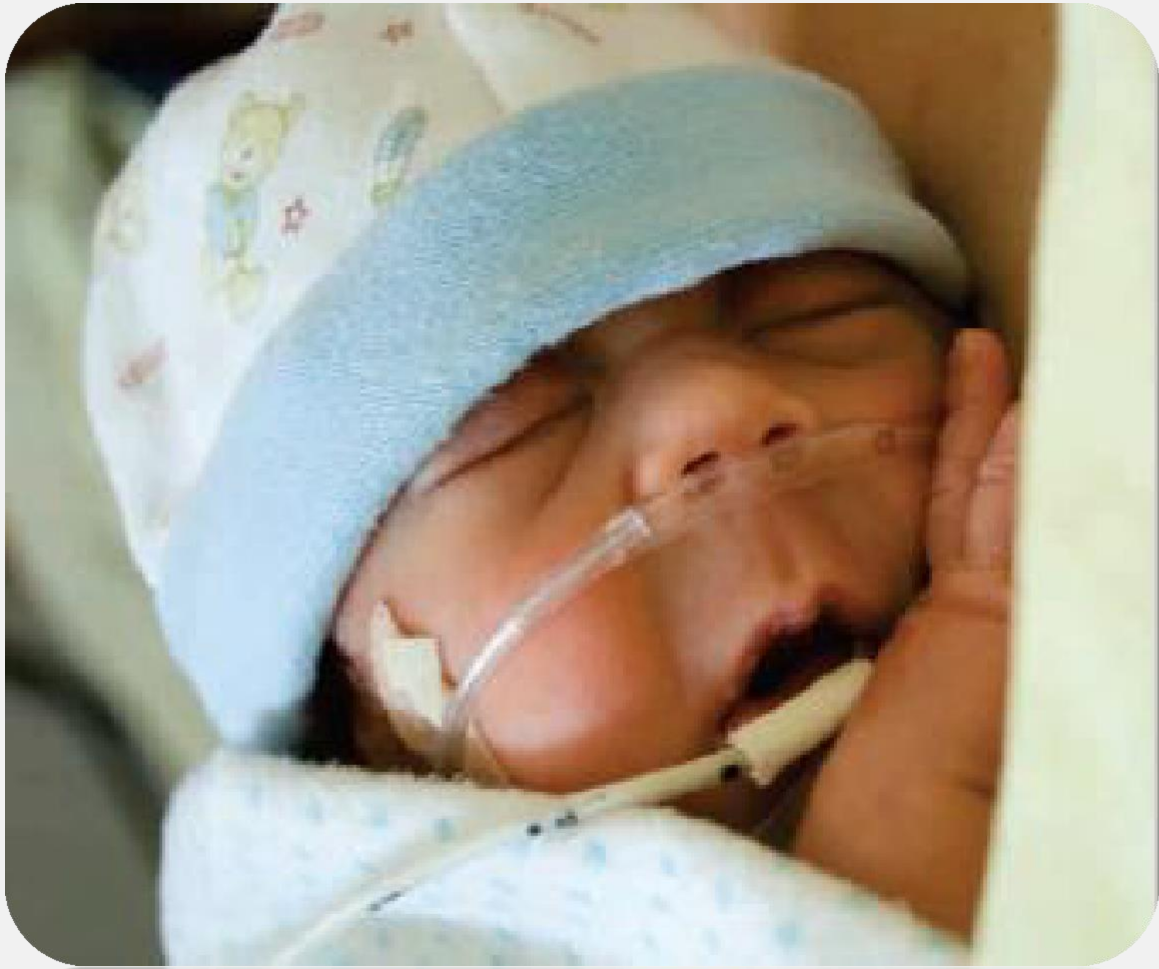

CPAP

(Continous Positive

Airway Pressure)

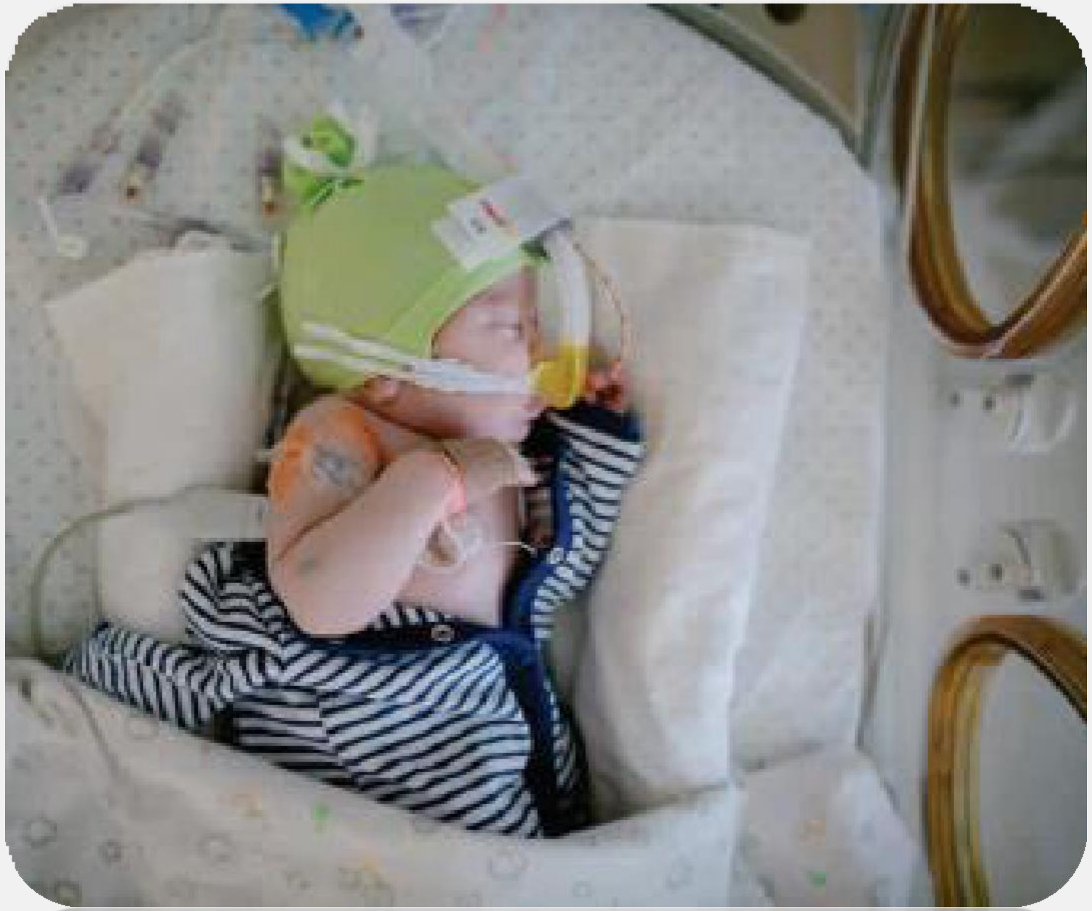

L'intubation et le tube endotrachéal

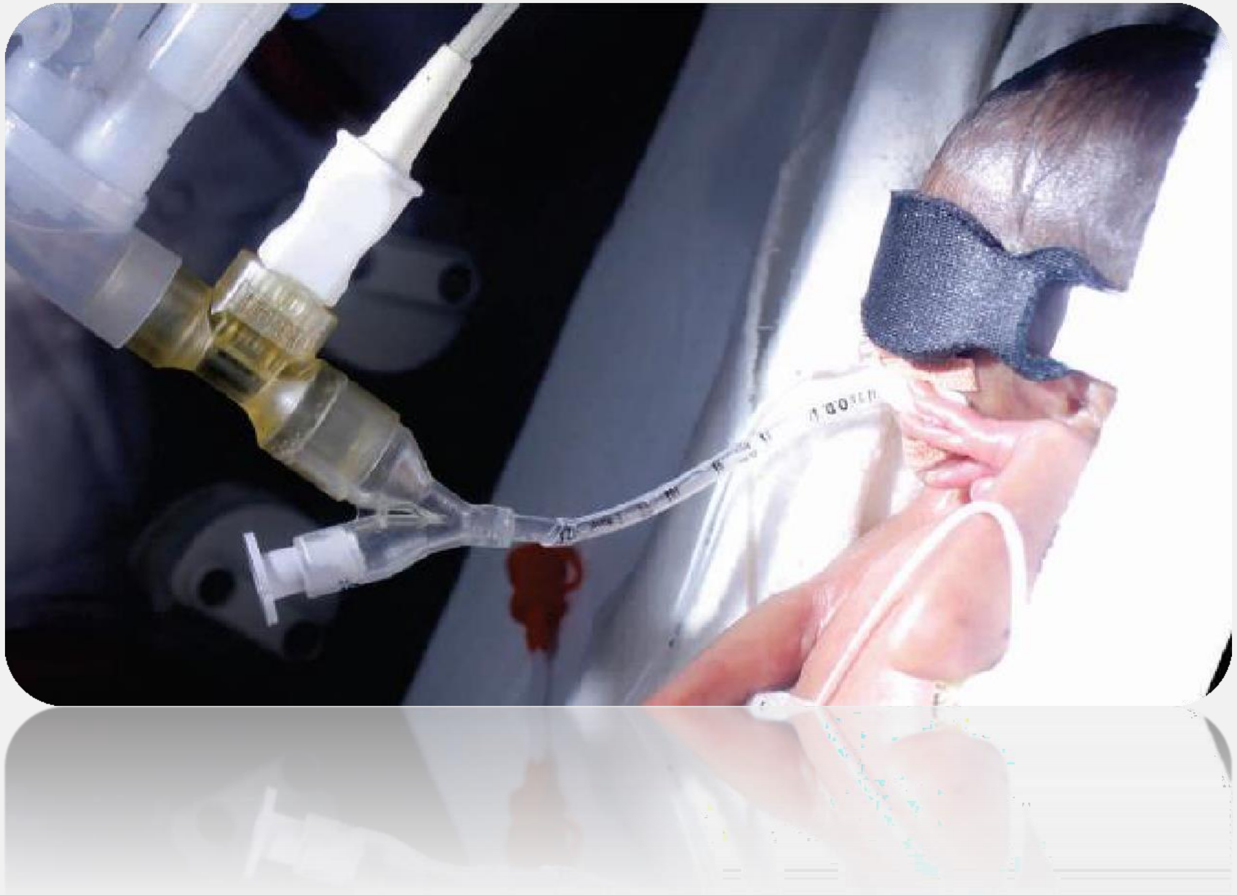

La place des parents

- **Vous êtes essentiels!**
- Le monde de la néonatologie est désorientant
- Accumulation de deuils et de rêves brisés
- C'est souvent un grand défi que d'apprendre à être parent, et en même temps apprendre comment aider son bébé
- Important d'y aller à votre rythme
- Sources de soutien: équipe de soutien fait partie de l'équipe

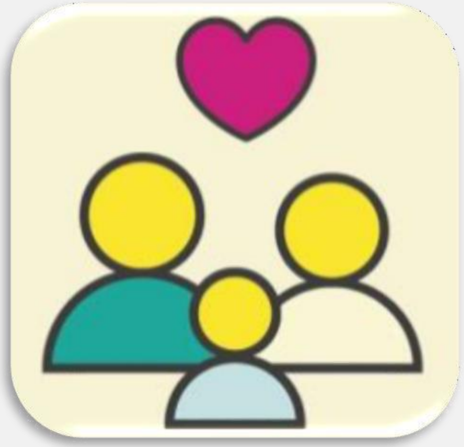

(psychologue, travailleuses sociales, intervenants spirituels)

- **Vous faites partie de l’équipe.**

De quelle façon pouvez-vous participer ?

- Les premières journées sont souvent les plus difficiles.
- Les parents, la famille et le bébé se découvrent tous les jours.
- Si maman ne peut pas venir voir son bébé durant les premières journées, des photos peuvent lui être montrées. Vous pouvez également placer une photographie de la famille au chevet du bébé.

Les interactions avec votre bébé

- **Même si votre bébé est petit ou fragile, vous pouvez le toucher et lui donner des soins:** changer sa couche, donner son bain, appliquer de la crème, changer les électrodes, etc.
- Il est important pour votre bébé d'entendre la voix de ses parents et sa famille, cela aide à développer son cerveau et ses sens. Parlez-lui doucement, appelez-le par son prénom, chantez-lui des chansons, lisez-lui un livre.
  - Programme musique et lecture.
- Les autres sens importants pour les bébés: l'odorat et le toucher

- **Prendre le bébé, lui parler, le bercer, c'est participer au développement de son cerveau.**

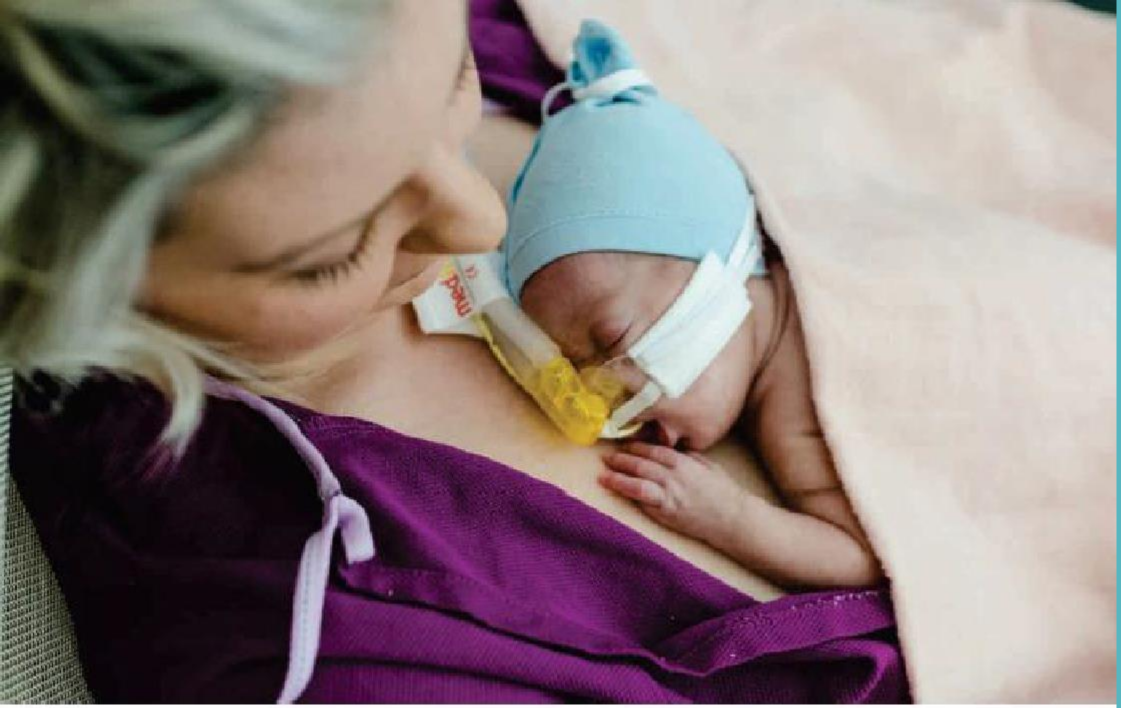

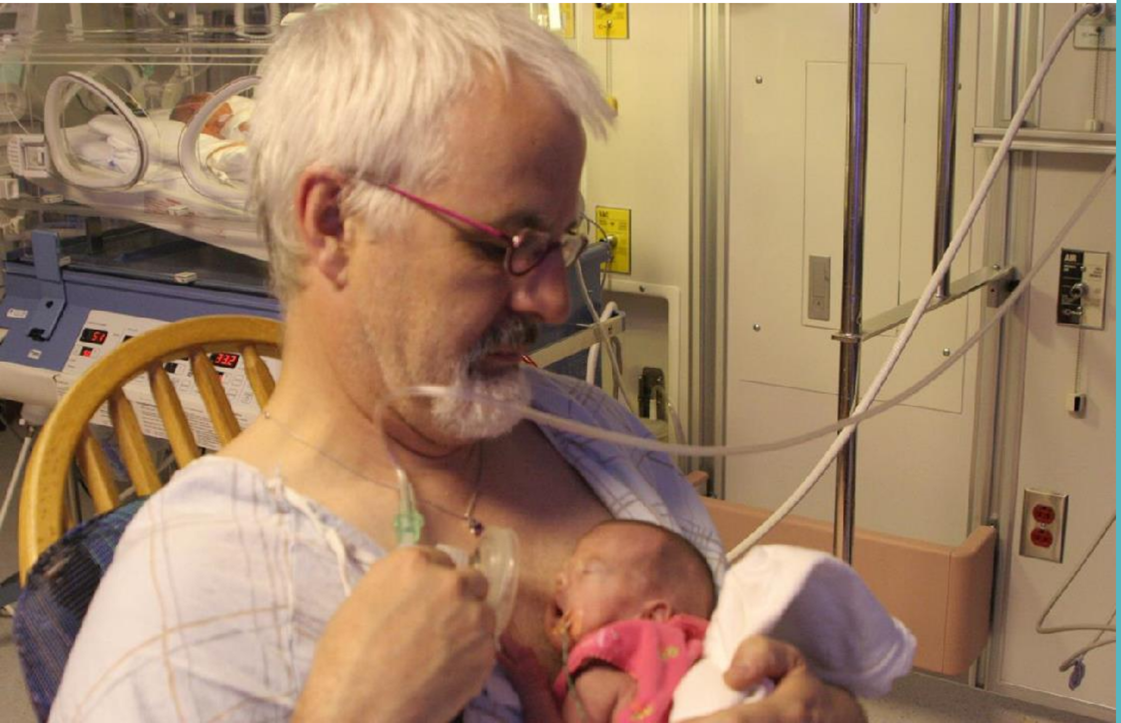

MÉTHODE KANGOUROU (LE PEAU-À-PEAU)

-Sensations agréables qui ressemblent à celles vécues dans le ventre de maman.

-Améliore la stabilité du bébé

-Facilite la production de lait pour maman

-Parfois, pour certains parents, il y quelques inquiétudes au début, mais ce moment reste souvent

La tournée médicale

- Tous les jours.
- Entre 10 h et 15 h, vous pouvez vous renseigner à votre infirmière afin de pouvoir être présents si vous le désirez.

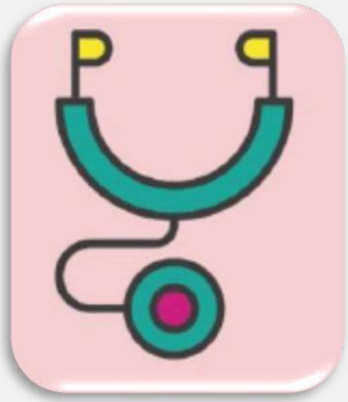

Un a leaublances avo

disposi1on dans cha u
sali i I s a 1 d 'un ou II de
com n1ca i vo s
1 s ro ess1onn 1s.

0 seil ter
0 sd 0 ..
pa.
au fur et à ms ure
d 0 bl1

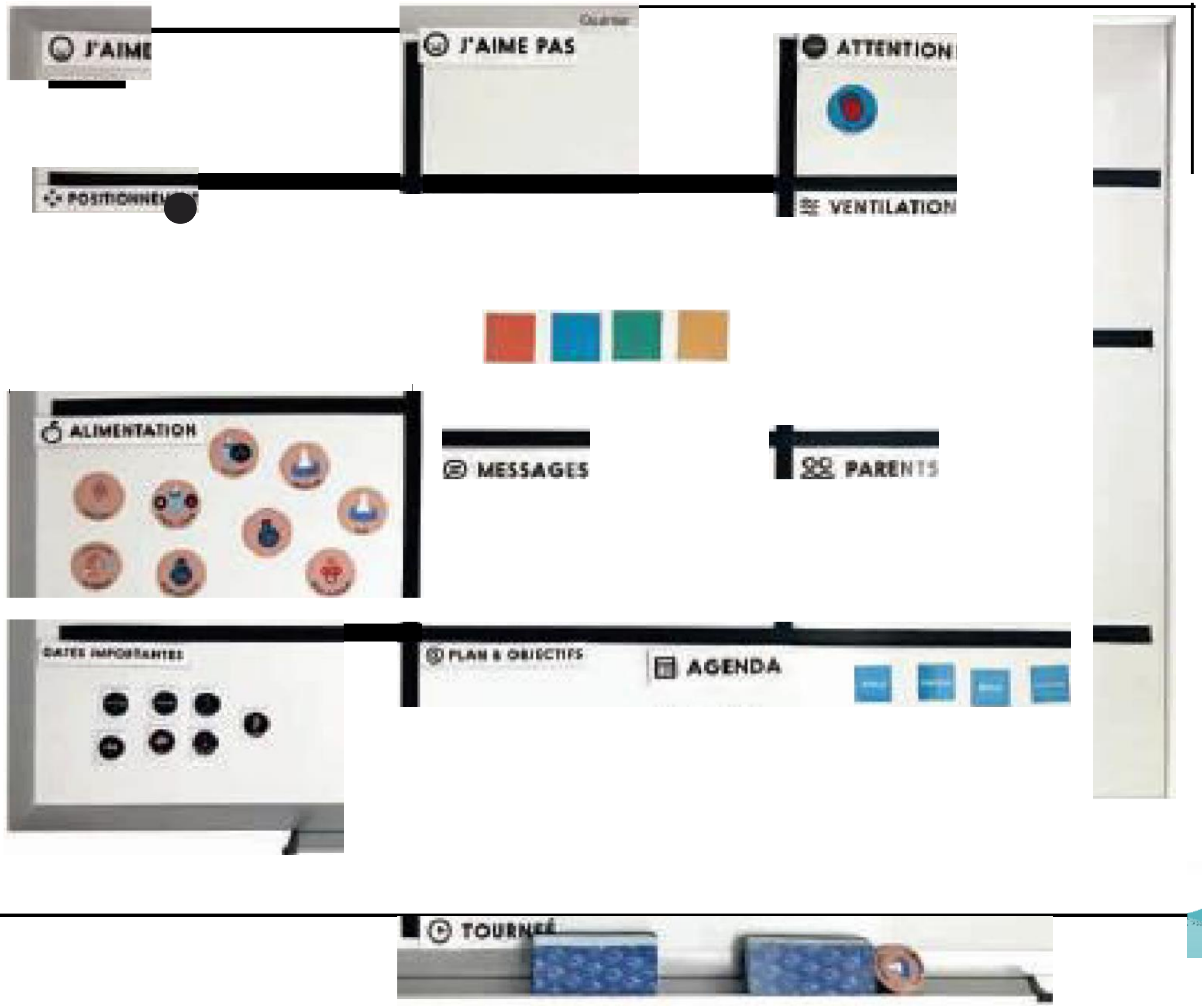

La nuit

- **Banquette-lit** disponible dans toutes les chambres.
- De nombreux parents décident de dormir à la maison. **Il est important de reprendre ses forces. Tous les parents sont différents, tous les choix sont bons.**
- Des services d'hébergement temporaire sont aussi disponibles pour les familles:

1. **Dans l'hôpital (Hôtellerie Chez Cachou)**
2. **À côté de l'hôpital (Manoir Ronald McDonald)**

LE CONGÉ DE LA NÉONATOLOGIE

- Possibilité pour certains bébés de retourner dans un centre plus près de leur domicile
- Congé à la maison a lieu quand bébé:
  - Capable de respirer et ne fait plus de pauses respiratoires
  - Capable de manger
  - Maintient sa température
  - Pas toujours à la date prévue d'accouchement, souvent

avant

Questions?

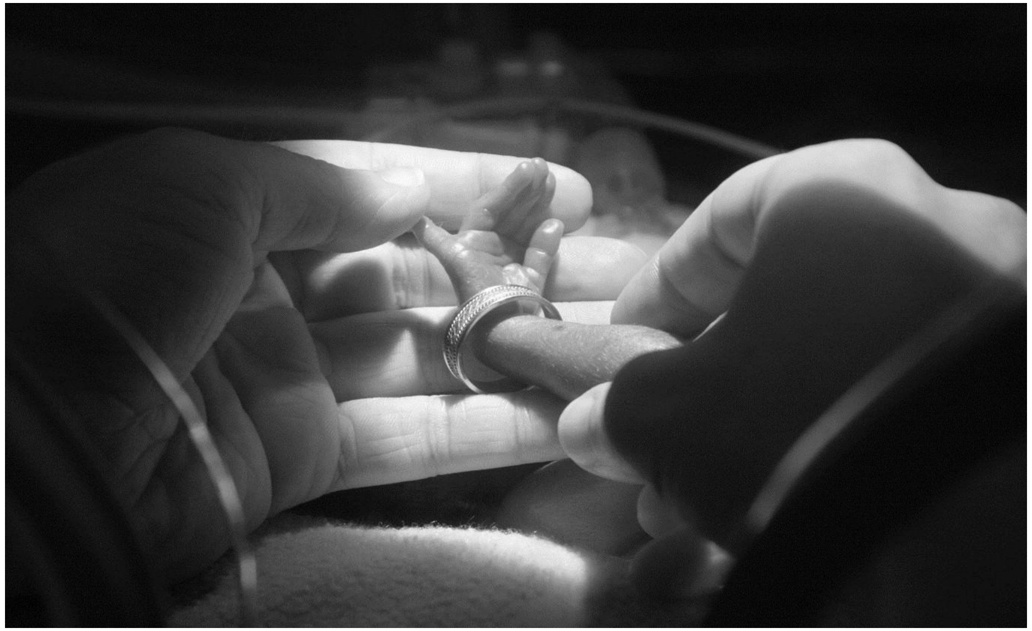

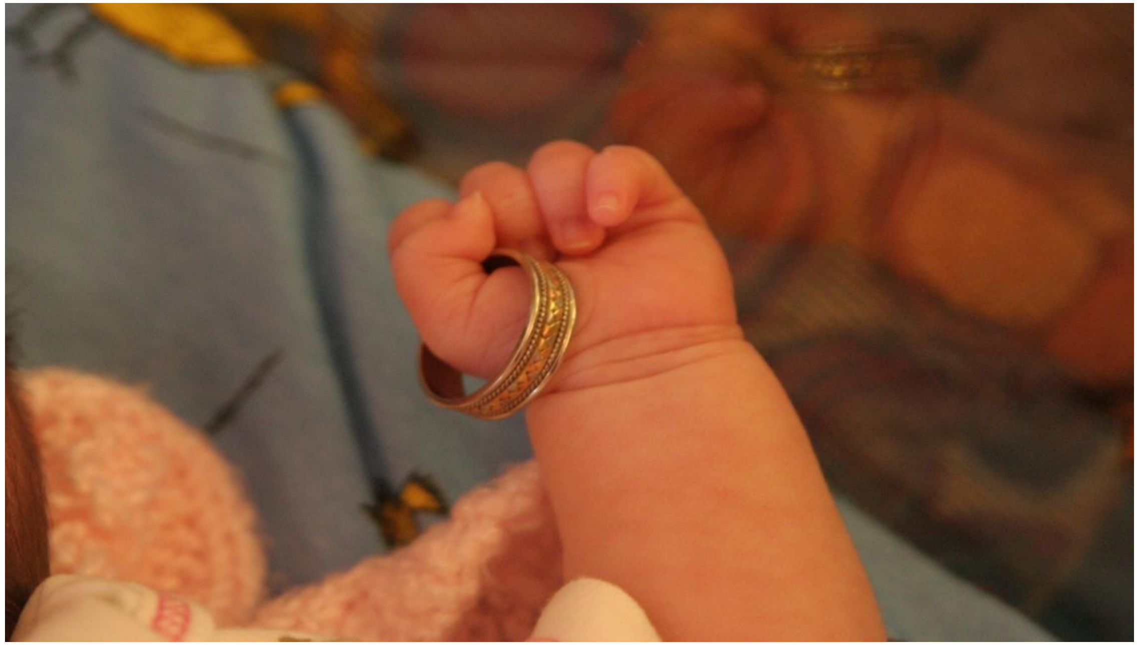

Supplement: Supplementary file 1 [file children-10-01570-s001.zip › children-2489210-supplementary.pdf]
